# Some Commonly Overlooked Causes for Stomach and Bowel Disturbances1Read before the American Therapeutic Society, May 8, 1908, Philadelphia

**Published:** 1908-10

**Authors:** Robert T. Morris

**Affiliations:** Professor of Surgery in the New York Post-Graduate Medical School and Hospital, etc.


### Some Commonly Overlooked Causes for Stomach and Bowel Disturbances.[1]

By ROBERT T. MORRIS, M. D.
Professor of Surgery in the New York Post-Graduate Medical School and Hospital, etc.

(*Monthly Cyclopedia*, August, 1908.)

If I were to give two points in advice to a class of medical students just about to begin lifework, I would say: first, keep out of debt; next, remember that cases of stomach and bowel trouble are not cases of stomach trouble. Most of the dyspepsias are nothing more than symptoms, like a cough or a sneeze. Why do the bowels cough and sneeze? Often because there is a little bit of pepper at the end of the cecum, known as the "appendix vermiformis," which happens to be undergoing infective or degenerative changes. This is one of the most commonly overlooked causes for stomach and bowel disturbance. Not at the time of an acute infective invasion with explosive features, when we have to dodge to get out of the way when the patient vomits, for we all recognise the character of the stomach and bowel disturbance right then; but if instead of an appendix undergoing infective invasion we have an appendix undergoing fibroid degeneration, we are apt to be looking the other way while the bowel coughs and sneezes mildly and chronically in what is known as intestinal dyspepsia. Instead of speaking of the appendix as a bit of pepper let us elaborate a little more and have two kinds of bowel exciters at this point: the appendix which is the seat of acute infective invasion is a sparker and keeps things going at a lively clip; the appendix undergoing fibroid degeneration, on the other hand, is a G. D. cap of the sort that we used to put on the old musket in the days when we loaded it up with slugs and watched behind the stone wall for a woodchuck. Sometimes the cap went, and sometimes it didn't; sometimes it just sizzled and fizzed. If we liken the appendix undergoing fibroid degeneration to a G. D. cap, we shall find that patients often refer to it the same way. We have, in this type of appendix, an irritative lesion rather than an infective lesion. It takes more patients to the doctors' office than any other kind of appendix trouble, but the doctors, in general, do not know it—this year. One doctor tells the patient that he has intestinal indigestion, and when the patient tires of this doctor he goes to another one who tells him the same thing, and that is about all the benefit the patient gets out of it. Why does the appendix undergoing fibroid degeneration cause so much disturbance and such persistent disturbance? It is because the coats of the appendix replaced by

1. Read before the American Therapeutic Society, May 8, 1908, Philadelphia connective tissue, retain their nerve filaments, and these nerve filaments are irritated by contracting connective tissue in the appendix, just as they are in the scar of an amputated leg. The irritated nerve filaments excite the intimate ganglia of the bowel wall (Auerbach's plexus and Meissner's plexus), and the function of the bowel is deranged. Sensory nerves as well as sympathetic nerves are excited, and these patients have a feeling of discomfort in the appendix region, which now and then amounts to actual pain, but it does not send the patient to bed. The patient is inclined to press over the appendix region with his hand; he suffers from chronic indigestion, and is in a state of more or less general unrest. It is my belief that this type of appendix, so common in middle life period, is a safeguard against real appendicitis, as we understand the term, and for two reasons: first, because the structures which are engaged in infective processes—mucosa, submucosa, and lymphoid—are being removed by connective tissue replacement; and, in the second place, because the irritation of entrapped nerve filament calls out a constant local hyperleucocytosis which is protective. This type of appendix is very different from the scarred appendix resulting from an acute infective invasion, because in the latter type nerves are destroyed along with other tissues, and we seldom have persistent stomach and bowel disturbance caused by the scarred appendices, unless peritoneal adhesions in the vicinity cause annoyance. So little is fibroid degeneration of the appendix known this spring, that many of these appendices have been operated upon by surgeons on the supposition that they were dealing with a chronic infection of the appendix, and surgeons operating in such cases have sometimes been so disappointed at finding nothing but a little degenerate appendix left, that they have closed the abdomen without removing it, and the patient has continued to suffer; whereas, if the surgeon had removed the little G. D. cap, his patient would have gained forty pounds in weight and would have had a feeling of deep gratitude for our whole profession.

These specimens which I pass about represent various stages of fibroid degeneration of the appendix, from specimens still retaining many of the normal structures, to specimens consisting of little more than fibrous strings, and yet each one of these specimens is the *memorabile* of a cured patient On account of my interest in the subject some of my assistants and friends got to calling this type of appendix by my name, but I object to this nomenclature for two reasons: first, because Senn and Ribbert both described the condition before I took up the study. Senn called the condition "appendicitis obliterans," and Ribbert referred to it as "a normal involution process." Senn's an "itis" that causes "obliterans," but an "obliterans" that causes the "itis." Ribbert's description is not comprehensive, because we do not associate involution with an inflammatory condition. Another reason why I do not wish to have these called Morris appendices, is because the nomenclature went wrong and got to stand for the normal appendix among many who quoted the name in a spirit of fun, and by *reductio ad absurdum* the logical deduction was that I believed in removing normal appendices. I have never at any time, in speaking or in writing, advocated the removal of normal appendices, and my reason is a very definite one. My reason is that in removing a normal appendix we open a tiny spigot of infection in a field that is not guarded by protective leucocytosis at the moment. If we remove an appendix which has been the seat of an infective process for a few hours, it makes little difference whether we wash our hands before or after operation, for the patient is protected; but when we remove a normal appendix in an unprotected field, look out for the little spigot!

Another commonly overlooked cause for stomach and bowel disturbance is eyestrain. Many ophthalmologists are familiar with the fact, but few are willing to give their time to a painstaking study of this particular part of their subject. They are too anxious to get to the next patient. San Jose scale is the worst pest in the apple orchard, and the "next patient" is the worst pest in the office of the ophthalmologist. It requires a great deal of special study on the part of the physician and surgeon to know which eye men should see into in their dyspepsia cases. Some ophthalmologists, without a proper sense of proportion, have made so much of the subject that their views are held to be fanciful and others have given so little attention to the subject that they are not qualified to express an opinion. Almost anyone of us can get from the patient a history of headaches or of frowns which will lead him to send his patient to the ophthalmologist with the request for either negative or positive testimony bearing upon the matter of dyspepsia.

Bile-tract adhesions cause an immense amount of stomach and bowel disturbance, and yet this cause is commonly overlooked. Most of us have adhesions in the region of the gallbladder and bile-ducts. These adhesions are commonly of no importance, and in other cases they are of dominating importance. Bacteria and their toxins taken from the bowel to the liver by the afferent vessels of the portal system, are in part excreted by the liver, and on the way out they set up inflammatory processes in the mucosa of the gallbladder and bile-ducts which lead to desquamation of endothelium on the peritoneal side, with exudation of plastic lymph. This lymph later forms adhesions which frequently engage the pylorus, the hepatic flexure in the colon, and other structures in the vicinity. This inflammation of the bile-tract region is often subacute, and passes by as many names as we formerly gave to appendicitis before we understood that condition.

Loose kidney is another commonly overlooked cause for stomach and bowel disturbance, and as with bile-tract adhesions there are loose kidneys which cause little or no disturbance, and there are other loose kidneys which act as a loose bolt in the patient's works. The discriminating diagnostician takes pains to classify the different kinds of loose kidney, and to apply tests which show the rôle played by loose kidney in any given case.

Ulcer of the stomach, obstruction of the circulation by nephritis, improper use of alcohol, ice water, and tea, are not included in my subject, because these causes for dyspepsia are not commonly overlooked. Diagnosticians are keen enough in making the diagnosis in this old field of observation, but according to my experience the recognised causes for dyspepsia are distinctly less in number than the overlooked causes. How are we to make a differential diagnosis between the different overlooked causes that I have brought forward today? In the first place, we will call up a central telephone station consisting of a pair of points situated approximately an inch and a half from the navel on either side of the navel. Many young business men complain that it is difficult to find a profitable occupation, and yet they are unwilling even to take the pains to select at the outset the kinds of occupation in which it is possible for them to become millionaires, but almost anyone can become a millionaire if he cares to take the trouble. In the same way almost anyone can make a diagnosis of the cause for almost any dyspepsia if he cares to take the trouble. Now, for a starting point, let us go to our pair of points which represent the lumbar ganglia, and which give testimony as soon as we ask for it, by making deep pressure with the fingers, to determine whether the right group or the left group is hyperesthetic, singly or together, or not at all. If we start out with examination of the pair of points, we shall find that this examination of the P. P. is of equal importance with urinary examination in older fields of work. Remember, that examination of the P. P. is of equal importance with examination of the urine. If the dyspepsia is caused by fibroid degeneration of the appendix, we shall find hyperesthesia at the point on the right side of the navel, singly and alone. If it is caused by sigmoiditis, we shall find the left group of ganglia hyperesthetic singly and alone. If it is caused by some pelvic condition, we shall find hyperesthesia at both right and left points; for instance, if an appendix and an oviduct happen to lie side by side in the pelvis, and we cannot determine by ordinary means which one is the source of irritation, call up the telephone station at the pair of points and find out. If it is the appendix, there will be hyperesthesia at the right point alone; if it is the oviduct, there will be hyperesthesia at both points. By making use of this telephone station we can often trace the cause for a dyspepsia to a hernia or to hemorrhoids, and I recently traced it in an obscure case to a distended left seminal vesicle that turned out to be a primary focus for tuberculosis, which is rare. If there is hyperesthesia at neither one of the pair of points, look for something above the level of the navel for the cause of the dyspepsia. Take up the question of eyestrain or of bile tract adhesions, or some of the commonly recognised causes for dyspepsia.

It is difficult for some of us, who are half a hundred years old, to take up a new line of observation in this way, and many men are so trained to the habit of resisting external impressions that they become convinced very slowly. I belong to this class myself, but I am very grateful to men with whom I disagree, and who oblige me finally to come to their points of view. The neurologists, as a class, are the hardest men to shift from their positions, because their knowledge is so weighty that it has ponderous momentum and velocity along established lines. Did you ever see a stag come to the spring at evening to drink? He steps out gracefully but boldly, elevates his beautiful head, shakes his magnificent antlers, and surveys the forest and glade and looks as though he owned it all. That is the neurologist. Do you know what a little 30.30 bullet behind his right shoulder-blade will do? This is the patient with nervous dyspepsia.

